# Risk-Reduction Research in Occupational Safety and Ergonomics: An Editorial

**DOI:** 10.3390/ijerph20065212

**Published:** 2023-03-16

**Authors:** Roger Jensen, David P. Gilkey

**Affiliations:** Department of Safety, Health, and Industrial Hygiene, Montana Technological University, Butte, MT 59701, USA

## 1. Introduction

Occupational health and safety is one of the pillars of public health. The magnitude of occupational injuries, illnesses, and fatalities has been well documented through retrospective analyses of existing record systems. This Special Issue in *IJERPH* sought research papers addressing risk reduction studies in occupational safety and ergonomics.

**Figure 1:**
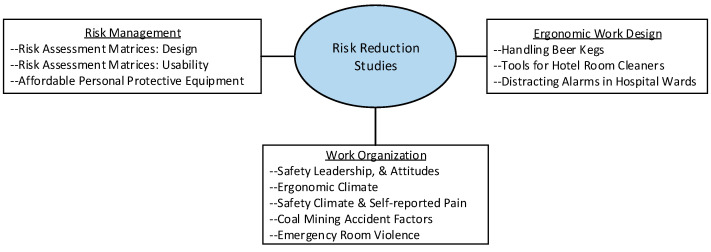
Depiction of how content of the papers in this Special Issue contributed to the body of knowledge regarding occupational safety and health.

All submissions were reviewed through the MDPI peer review process, resulting in the acceptance and publication of eleven research papers. The studies contributed to the body of knowledge in risk management approaches, work organization, and ergonomic work design as depicted in Figure 1.

## 2. Risk Management Approaches

Regarding risk management approaches, two papers contributed to the design of risk assessment matrices [1,2]. The first study addressed the terms used to define ordered categories of the two axes of most risk assessment matrices—severity and likelihood. Data for recommending particular sets of terms were initially obtained by surveying undergraduate students majoring in occupational safety and health [1]. A follow-on survey of graduate students studying industrial hygiene also provided ratings of terms for severity and likelihood [2]. The results of the second survey confirmed those of the first survey with a few modifications. The second paper provided insights into less recognized aspects of risk assessment matrices including both qualitative and quantitative approaches for determining risk indicators and using research-based terms to enhance usability for risk assessment teams as they estimate the severity and likelihood of future accidents.

The third paper reported an exploratory study addressing the exposure to pesticide of agricultural workers in Thailand [3]. According to the study authors, many workers apply insecticides without chemical protective clothing due to the cost of the full-suit personal protective equipment that meets international standards. The study reported the findings of a laboratory examination of a protective suit made with a low-cost material.

## 3. Work Organization Approaches

Regarding work organization approaches, five papers contributed to the body of knowledge [4,5,6,7,8]. One of these papers described findings from surveys involving construction workers carrying out electrical substation projects in Saudi Arabia [4]. The researchers used the survey results to explore the relationship between factors related to organizational climate and the perceived influence on safety. Additionally, the authors used the survey results to propose a graphic model depicting the relationships between seven factors: (1) safety leadership, (2) safety attitudes, (3) the interaction between factors 1 and 2, (4) safety motivation, (5) safety knowledge, (6) safety participation, and (7) safety compliance. The Special Issue editors expect the graphic model will become the most cited outcome of the paper.

The second survey addressing organizational approaches to occupational safety obtained inputs from employees at two power plants in Iran [5]. The 42-item survey included items reflecting self-reported pain, performance, and wellbeing. Using data from 109 of 110 employees, the researchers explored the relationships between ergonomic climate factors and self-reported musculoskeletal symptoms [5]. One of the two main findings was that the respondents who reported experiencing musculoskeletal pain had significantly higher scores on the ergonomic climate survey.

The third survey involving work organization involved 459 employees working in the United States stone, sand, and gravel industry [6]. The survey asked about the signs and symptoms of musculoskeletal conditions. The prevalence of these conditions was compared to responses about processes used for ergonomic hazard identification and weekly hours of work. High prevalence rates of reported musculoskeletal pain were found among those working as a mechanic/maintenance worker. Higher prevalence rates were found among those working more than 60 h per week. The study authors recommend improving methods for ergonomic hazard identification and limiting weekly work hours.

The fourth paper on work organization reported findings based on analyses of coal mining accident investigation reports in China [7]. The researchers performed sophisticated analyses to find organizational factors associated with the accidents [7]. The analysis involved starting with 883 accident reports based on phrases found in the reports. These were categorized into 55 manifestations suggesting causal contributors to the accidents. The authors sorted these factors based on the human factors analysis and classification system (HFACS). The five categories, from most distal to most proximal, were: external influences, organizational influences, unsafe supervision, unsafe preconditions, and unsafe acts. Based on these analyses, the authors presented a coal mining version of the HFACS which they refer to as HFACS-CM [7].

A fifth survey related to work climate examined hospital emergency room violence experienced by hospital employees [8]. The survey described four incident scenarios and asked respondents if they considered the incident a reportable crime. The survey revealed differences between hospital staff and the local police regarding what incidents should be reported and how to account for patients with various mental conditions. Hindsight suggests the term “reportable crime” was understood by police as a specific criminal act whereas hospital personnel may have been unclear if “reportable” meant reportable to local police or reportable within the hospital organization. Perhaps the main contribution of the survey results is for future research on this topic to use more specific items on violence reporting to learn how hospitals might integrate patients’ mental state into reporting policies.

## 4. Ergonomic Work Design

Regarding ergonomic work design approaches, two papers described comparisons between a current work method with a more ergonomic method proposed by the study authors [9,10]. The authors of the first of these papers applied biomechanical analysis to a task involving the manual handling of beer kegs in a brewery [9]. Using wearable technology, the investigators used results of their biomechanical analyses to identify a less stressful work arrangement for handling the beer kegs [9]. The authors of the second paper examined posture-stressing tasks required of hotel room cleaners., and an intervention involving long-handled cleaning tools was successfully tested [10].

Another survey-related study described how to measure the effects of frequent patient alarms in hospital intensive care units [11]. The authors sought to translate a survey originally established in English into the Polish language. The process involved having an initial translation from English to Polish (forward translation) followed by the Polish language version being back-translated to English by different translators. The processes also included reviews for possible cultural differences using staff who regularly work in a Polish intensive care unit. The resulting Polish language survey instrument will provide a basis for future studies into the influence of frequent alarms in ICUs and some other hospital areas. The study also illustrated the complexity involved in translating a standardized questionnaire from the original language to a different language.

## 5. Conclusions

In conclusion, we would have appreciated more submissions for our Special Issue, but we are satisfied with slightly exceeding our initial goal of ten. The papers represent some of the more common approaches to occupational safety research. The set of eleven has three on risk management, five on work organization, and three on ergonomic work design. The international nature of safety research is represented by the countries of the principal authors: Thailand [3], Saudi Arabia [4], Iran [5], China [7], Poland [11], and the United States [1,2,6,8,9,10].

## References

[1] Jensen R.C., Hansen H. (2020). Selecting appropriate words for naming the rows and columns of risk assessment matrices. Int. J. Environ. Res. Public Health.

[2] Jensen R.C., Bird R.L., Nichols B.W. (2022). Risk assessment matrices for workplace hazards: Design for usability. Int. J. Environ. Res. Public Health.

[3] Naksata M., Watcharapasorn A., Hongsibsong S., Sapbamrer R. (2020). Development of personal protective clothing for reducing exposure to insecticides in pesticide application. Int. J. Environ. Res. Public Health.

[4] Basahel A.M. (2021). Safety leadership, safety attitudes, safety knowledge and motivation toward safety-related behaviors in electrical substation construction projects. Int. J. Environ. Res. Public Health.

[5] Faez E., Zakerian S.A., Azam K., Hancock K., Rosecrance J. (2021). An assessment of ergonomics climate and its association with self-reported pain, organizational performance and employee well-being. Int. J. Environ. Res. Public Health.

[6] Balogun A.O., Smith T.D. (2020). Musculoskeletal symptoms among stone, sand, and gravel mine workers and associations with sociodemographic and job-related factors. Int. J. Environ. Res. Public Health.

[7] Fa Z., Li X., Liu Q., Qiu Z., Zhai Z. (2021). Correlation in causality: A progressive study of hierarchical relations within human and organizational factors in coal mine accidents. Int. J. Environ. Res. Public Health.

[8] McGuire S.S., Mullan A.F., Clements C.M. (2022). Workplace violence in the emergency department: Case study on staff and law enforcement disagreement on reportable crimes. Int. J. Environ. Res. Public Health.

[9] Brents C., Hischke M., Reiser R., Rosecrance J. (2021). Trunk posture during manual materials handling of beer kegs. Int. J. Environ. Res. Public Health.

[10] Allread W.G., Vossenas P.V. (2022). Comparisons of trunk motions and low back injury risk between alternative hotel cleaning methods. Int. J. Environ. Res. Public Health.

[11] Rypiez L., Rozensztrauch A., Fedorowicz O., Włodarczyk A., Zatońska K., Juárez-Vala R., Witczak I. (2023). Polish adaptation of the Alarm Fatigue Assessment Questionnaire as an element of improving patient safety. Int. J. Environ. Res. Public Health.

